# Traditional Classification and Novel Subtyping Systems for Bladder Cancer

**DOI:** 10.3389/fonc.2020.00102

**Published:** 2020-02-07

**Authors:** Shaoming Zhu, Weimin Yu, Xiao Yang, Cheng Wu, Fan Cheng

**Affiliations:** ^1^Department of Urology, Renmin Hospital of Wuhan University, Wuhan, China; ^2^Department of Gynaecology and Obstetrics, Renmin Hospital of Peking University, Beijing, China

**Keywords:** bladder cancer, molecular subtypes, EOTRC, clinical prognosis, multiomics

## Abstract

Bladder cancer is the most common tumor in the urinary system, with approximately 420,000 new cases and 160,000 deaths per year. The European Organization for Research and Treatment of Cancer (EOTRC) classifies non-muscular invasive bladder cancer (NMIBC) into low-risk, medium-risk and high-risk groups based on a comprehensive analysis of NMIBC pathological parameters and the risk of recurrence and progression to muscular invasive bladder cancer (MIBC). Traditional classification systems are based on pathologic grading, staging systems, and clinical prognosis. However, the pathological parameters of the tumor cannot fully reflect the “intrinsic characteristics” of bladder cancer, and tumors with a similar pathology exhibit different biological behaviors. Furthermore, although the traditional classification system cannot accurately predict the risk of recurrence or the progression of bladder cancer patients (BCs) individually, this method is widely used in clinical practice because of its convenient operation. With the development of sequencing and other technologies, the genetics-based molecular subtyping of bladder cancer has become increasingly studied. Compared with the traditional classification system, it provides more abundant tumor biological information and is expected to assist or even replace the traditional typing system in the future.

## Introduction

Bladder cancer (BC) is the most common urinary tract tumor, with approximately 420,000 new cases and 160,000 deaths per year, and the incidence ratio of men:women ranges from 6:1 to 2:1 (1, 2). There are many risk factors for bladder cancer, including occupational and environmental factors [metal processing, dyes, rubber, plastics and exposure to other aromatic compounds (3)], living environment factors (arsenic in water, chlorine disinfectants, and nitrate) (2, 3), personal/dietary factors [coffee, smoking (4)], infection of pathogenic microorganisms (schistosomiasis and urinary tract bacteria) (2, 5), drugs (pioglitazone was recently shown to not be significantly associated with the risk of bladder cancer), patient age, gender, race, and socioeconomic status (2).

Bladder cancer is divided into non-muscular invasive bladder cancer (NMIBC) and muscular invasive bladder cancer (MIBC) according to the European Association of Urology (EAU) guidelines. Bladder cancer can also be divided into several types—urothelial carcinoma, squamous epithelial carcinoma, and adenocarcinoma—among which over 90% are urothelial carcinomas (6). In 2006, Sylvester et al. comprehensively analyzed the pathological parameters and risk of recurrence and progression of 2,596 NMIBC patients (NMIBCs) with Ta and T1 stage bladder cancer (7). The risk of recurrence and progression can be directly calculated and predicted by the pathological parameters in the corresponding software system (https://www.eortc.be/tools/bladdercalculator/). The European Organization for Research and Treatment of Cancer (EORTC) prediction system is mainly based on the following pathological parameters: previous recurrence rate (primary, recurrence ≤ 1 per year, recurrence > 1 per year), number (1, 2–7, 8 or more), diameter (<3 cm, ≥3 cm), T stage (Ta, T1), grading (WHO 1973) (grade 1, 2, 3), and whether or not it is combined with carcinoma *in situ* (CIS). According to the differences among these pathological parameters, recurrence and progression, EAU guidelines further classify NMIBC into low-risk, medium-risk and high-risk groups (6). Due to the classification system, pathological information is easy to obtain, the system is simple to operate, and the prediction is relatively accurate; therefore, it is widely used in the clinic.

## Traditional Classification and Molecular Subtyping Systems

The traditional classification system for bladder cancer is mainly based on pathological parameters (8, 9). Under similar pathological staging and grading, the recurrence and progression of bladder cancer vary greatly among different individuals and directly affect the optimal monitoring and treatment schedules (10, 11). For example, according to the EORTC software system calculation (https://www.eortc.be/tools/bladdercalculator/), primary Ta grade 3 bladder cancer is a high-risk bladder cancer, with a 5 years recurrence rate of 46% and a 5 years progression risk of 6%, which indicates that 94% of patients will not progress to MIBC within 5 years. Thus, the following question arises: why are all patients monitored and treated with the same program? Despite their similar pathological parameters, the biological behaviors of tumors are quite different. Some patients were pathologically classified as low-risk, but the actual tumor showed highly invasive biological characteristics and early metastasis, leading to the following question: can conventional conservative treatment be administered to all low-risk patients? Some high-risk patients do not respond to BCG, while others are sensitive to BCG (Bacillus Calmette-Guerin) (12, 13). However, the traditional classification system cannot predict this response. Therefore, it is difficult for doctors to choose schedules for BCG perfusion, radical cystectomy or another treatment according to the traditional prediction system. Some tumors are less likely to metastasize and need only local resection, while other tumors are highly invasive and need radical cystectomy and/or another treatment. Unfortunately, there is still no effective means to distinguish the two. Moreover, the traditional classification system can only predict the risk of recurrence and progression of NMIBC but not the risk of MIBC. After radical cystectomy, some muscle invasive bladder cancer patients (MIBCs) benefit from neoadjuvant chemotherapy (NAC), while others do not (14–16), which cannot be predicted by the traditional classification system. The pathological parameters of the tumor cannot fully reflect the “intrinsic characteristics” of bladder cancer. Therefore, it is difficult for doctors to provide individual and precise supervision and treatment for bladder cancer.

With the rapid development of sequencing, mass spectrometry and other techniques, studies based on genomics and transcriptome (17), epigenetics (18), proteomics (19) and other omics (20–22) will provide a new direction for the accurate diagnosis and treatment of bladder cancer. A good classification system should meet the following criteria: I. It should provide an accurate prediction for the risk of recurrence and progression of bladder cancer and help develop individualized monitoring, conservative treatment, surgical schemes, adjuvant therapies and follow-up schedules. II. It should be able to accurately identify individuals with tolerance to chemotherapy, targeted therapy, immunotherapy and help develop individualized adjuvant treatment plans. III. It should provide not only important information for predicting the recurrence and prognosis of bladder cancer but also effective molecular information for the study of molecular mechanisms, such as tumor development, progression, chemotherapy and immunotherapy tolerance. IV. It should also aid in the development of personalized new molecular diagnoses and treatments. In particular, the study of molecular subtyping based on genetics has made rapid progress in the study of bladder cancer and has attracted increasing attention (23–25). The development of molecular subtyping based on genomics and transcriptomes, etc., provides a new way to understand bladder cancer.

**Figure 1:**
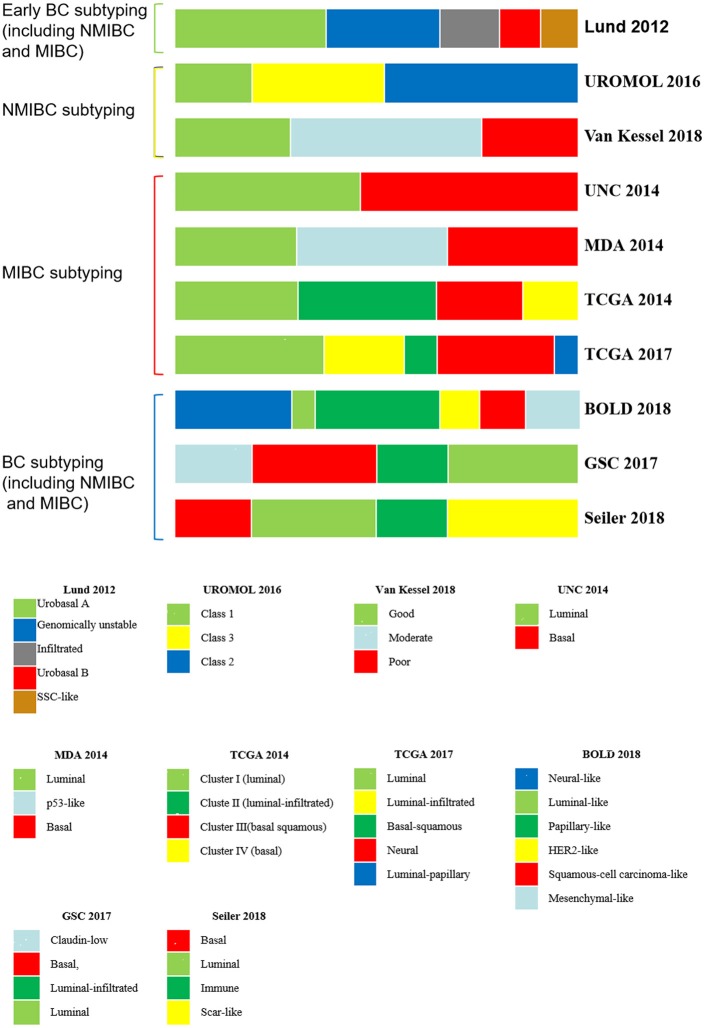
BC molecular subtyping systems. BC, bladder cancer; NMIBC, non-muscular invasive bladder cancer; MIBC muscular invasive bladder cancer.

Molecular subtyping was not first applied in the study of bladder cancer; initially, molecular subtyping was widely conducted in acute leukemia (26), breast cancer (27), colorectal cancer (28), hepatocellular and intrahepatic cholangiocarcinoma (29), and gastric cancer (30). The basic method of molecular subtyping is to detect gene expression in tumor tissues by sequencing, microarray and other technologies and then perform a cluster analysis of the different genes based on the gene expression level and genes involved in the biological process. Eventually, BCs are divided into several subtypes, and different molecular subtypes reflect different intrinsic and clinical characteristics of the tumors. For example, patients with basal-like or *HER-2*-enriched breast tumors have a poor clinical prognosis (31). Although patients with basal-like or *HER-2*-enriched breast cancer have a poor clinical prognosis, they are sensitive to neoadjuvant chemotherapy (NAC), which can benefit patients with this subtype (31). For the two major types of bladder cancer, NMIBC and MIBC, the molecular subtyping of bladder cancer can be divided into early BC subtyping, NMIBC subtyping, MIBC subtyping, etc. (Table 1; Figure 1).

**Table 1:** Major molecular subtyping systems.

**Subtyping systems**	**Abbreviation**	**Cases of the studies**	**Subtypes**
Early BC subtyping (including NMIBC and MIBC)	Lund 2012 five subtyping system (32)	*n* = 308	Urobasal A, genomically unstable, infiltrated, urobasal B, and SSC-like
NMIBC subtyping	UROMOL 2016 three subtyping system (33)	*n* = 460	Class 1, Class 3, and Class 2
	Van Kessel et al. (34)	*n* = 1,239	Reclassify EAU high-risk NMIBCs into good, moderate, and poor subtypes
MIBC subtyping	UNC 2014 two subtyping system (31)	*n* = 262	Basal-like (including claudin-low), and luminal
	MDA 2014 three subtyping system (35)	*n* = 73	Luminal, p53-like, basal
	TCGA 2014 four subtyping system (36)	*n* = 129	Clusters I, II, III, and IV (Luminal, luminal-infiltrated, basal squamous, basal)
	TCGA 2017 five subtyping system (37)	*n* = 412	Luminal, luminal-infiltrated, basal-squamous, neural, and luminal-papillary
BC subtyping (including NMIBC and MIBC)	BOLD 2018 six subtyping system (38)	*n* = 2411	Neural-like, luminal-like, papillary-like, HER2-like, Squamous-cell carcinoma-like and mesenchymal-like
Subtyping based on adjuvant chemotherapy	GSC 2017 four subtyping system (39)	*n* = 343	Claudin-low, basal, luminal-infiltrated, and luminal
	Seiler 2018 four subtyping system (40)	*n* = 116	CC1, CC2, CC3, and CC4 (Basal, luminal, immune, scar-like)

*BC, Bladder cancer; NMIBC, non-muscle invasive bladder cancer; MIBC, muscle invasive bladder cancer; SSC, squamous cell carcinoma; GSC, single-sample genomic subtyping classifier; CC, consensus clusters*.

## Early Molecular Subtyping of Bc

### Lund 2012 Five Subtyping System

**Figure 2:**
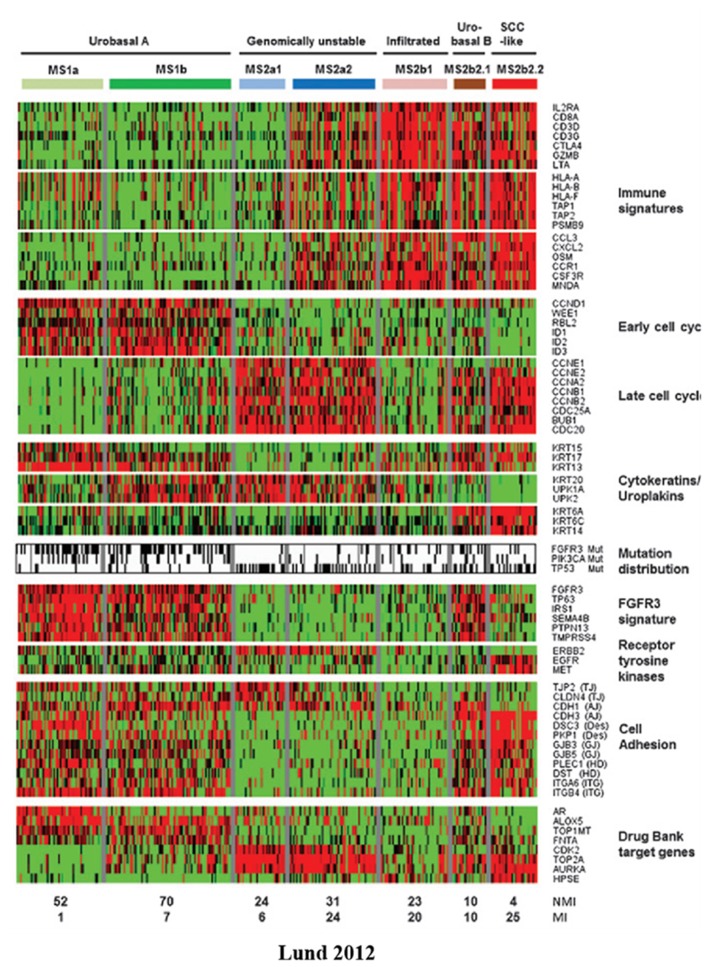
Heatmap of mRNA-seq data from the Lun 2012 molecular subtyping system. BCs are divided into five subtypes according to their genetic expression profiles (32). Red, high expression; green, low expression; black; mutation; white, wild-type; gray, no mutation data. SSC, squamous cell carcinoma; FGFR3, fibroblast growth factor receptor 3.

The earliest representative study on the molecular subtyping of bladder cancer was conducted by Sjodahl et al. at the University of Lund in 2012 (Lund 2012) (32). In this study, the transcriptome data of 308 bladder cancer samples were collected to analyze the expression levels of 13,953 genes, the biological processes involved in these genes (including immunological characteristics, cell cycle, cytokeratins/uroplakins, FGFR3 characteristics, etc.), and mutations in the *FGFR3, PIK3CA*, and *TP53* genes. BC was divided into five subtypes: urobasal A, genomically unstable, urobasal B, squamous cell carcinoma like (SSC-like), and infiltrated (Table 1). The urobasal A subtype exhibits an elevated expression of genes related to the early cell cycle, uroplakins, the FGFR3 signature, cell adhesion and other biological processes, while the genomically unstable subtype is characterized by immune characteristics and an elevated expression of genes related to the late cell cycle. The urobasal B, SSC-like, and infiltrated subtypes all highly expressed immunological signature genes, whereas the urobasal B and SSC-like subtypes highly expressed genes involved in the late cell cycle and cell adhesion (Figure 2).

An analysis of the prognoses associated with the five subtypes revealed that the prognoses of the five subtypes were significantly different. The urobasal A and infiltrated subtypes have a relatively better prognosis, while the SSC-like and urobasal B subtypes have the worst prognosis. By subtyping the tumors classified as grade 3, it was found that urobasal A has the best prognosis, while urobasal B has the worst prognosis, suggesting that even in the same pathological classification, clinical characteristics differ from their intrinsic molecular subtypes. The authors further explored the relationship between molecular subtypes and histopathologic classification and found that tumors in the Ta stage are mainly composed of the urobasal A subtype. In the T1 stage, tumors are mainly composed of urobasal A and genomically unstable subtypes. All subtypes of MIBC account for a certain proportion. Urobasal A is the main tumor subtype in G1 and G2 BC. G3 is similar to MIBC in that all subtypes account for a certain proportion. The above results indicate that there is a certain consistency between the molecular subtypes and pathological classification. In tumors with good stages and grades, the urobasal A subtype is the main component, while in those with a poor clinical prognosis, the proportion of the urobasal A subtype decreases significantly, and all subtypes account for a certain proportion.

## Molecular Subtyping of Nmibc

Compared with the number of MIBC molecular subtyping studies, the number of NMIBC molecular subtyping studies is low, which may be because partial bladder cancer specimens are not easy to obtain, especially Ta BC, with single and small-sized tumors. Therefore, there may be some missing data, resulting in publication bias. Representative studies include UROMOL 2016 subtyping (33) and Van Kessel 2018 high-risk NMIBC subtyping (34). Thus, far, these two studies have the largest number of experimental cases in NMIBC molecular subtyping studies.

### UROMOL 2016 Three Subtyping System

Hedegaard et al. (33) performed a transcriptome sequencing analysis of 476 samples (460 NMIBC, 16 MIBC) from multiple centers. Based on the expression levels of 8,074 genes and biological processes involved in the early cell cycle, the late cell cycle, keratins, uroplakins, epithelial-mesenchymal transformation, differentiation-related genes, etc., NMIBC patients can be classified into three categories according to the results of the cluster analysis: Class 1, Class 3, and Class 2. The Class 1 subtype highly expresses early cell cycle-related genes, and the clinical prognosis is the best. The late cell cycle-related genes of the Class 2 subtype are highly expressed, and the clinical prognosis is poor. The Class 1 and Class 2 subtypes both highly expressed uroplakins, while Class 3 shows undifferentiation. In addition, by analyzing the genes of bladder cancer stem cell markers, it was found that Class 3 is enriched in stem cells and the basal cell marker *CD44* gene. Compared with Class 1 and Class 3, Class 2 is enriched in EMT (epithelial-mesenchymal transition)-related genes.

**Figure 3:**
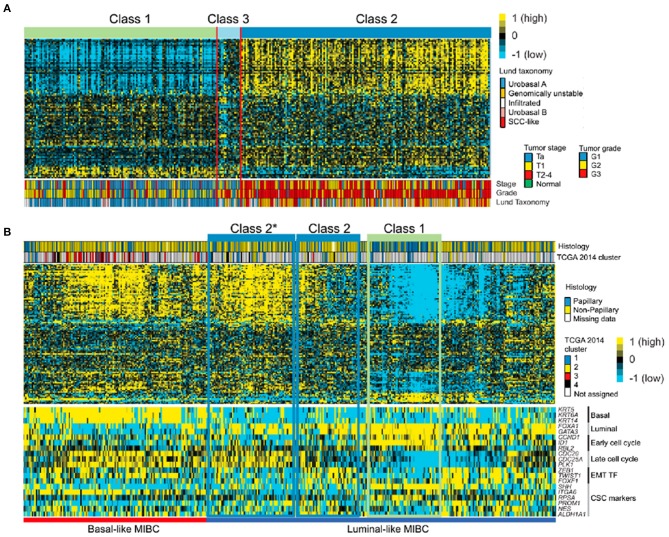
The correlation among UROMOL 2016 and other molecular subtyping systems (33). **(A)** The UROMOL 2016 subytping system analyzed the microarray data from 306 tumors from the Lund group based on 95 of 117 classifier genes (33). The Class 1 subtype is highly similar to the urobasal A subtype, and Class 1 subtypes mainly consist of low-risk NMIBC, while high-risk NMIBC tends to be concentrated in the Class 3 and Class 2 subtypes. **(B)** UROMOL 2016 was used to subtype the TCGA database (33). Heatmap of mRNA-seq data from 408 MIBC samples from TCGA based on 107 of the 117 classifier genes. Class 1 and Class 2 tumors are highlighted based on expression profile similarities to original classes. Class 2*: tumors with high similarity to Class 2 but with differences in EMT and CSC marker expression (33). Yellow, high expression; blue, low expression. SSC, squamous cell carcinoma; EMT, endothelial-mesenchymal transformation; CSC: cancer stem cell.

The team used 117 major genes from the subtype analysis to classify 130 BCs (85 Ta, 37 T1, 8 MIBC) and eight normal samples and analyzed the relationship between the molecular subtyping system and pathological classification. High-risk bladder cancer with a high stage and a high grade mainly consists of Class 2 and 3 subtypes. The main components of normal or Ta BC are Class 1, and the Class 2 subtype is most likely to progress to MIBC. Subsequently, these 117 genes were used to classify the data of 306 NMIBCs in the Lund 2012 study. It was found that 306 NMIBCs are mainly composed of Classes 1 and 2, and only 5% are composed of Class 3. The team also analyzed 408 MIBCs in the TCGA (The Cancer Genome Atlas) database, and the Class 3 subtype was not found. The above results suggest that the Class 3 subtype mainly exists in the NMIBC group, which is less than that of Class 1 and Class 2, and has a poor clinical prognosis. Class 1 is similar to the urobasal A subtype in the Lund 2012 system and the papillary subtype in the MIBC TCGA 2014 system (Figure 3).

The team further analyzed the genomic mutations and found that the median number of single nucleotide polymorphisms (SNPs) per patient was 218 (ranging from 1 to 863). The most common mutations were *PIK3CA* (*n* = 108) and *FGFR3* (*n* = 279). The mutated genes are mainly related to cell structure, cytoskeletal function, chromatin organization and chromatin remodeling. Chromatin remodeling gene mutations were found in 86% of the samples, mainly in the Class 2 subtype, and were concentrated in DNA damage repair, *MAPK*/*ERK*, and *ERBB* family genes. *APOBEC* family gene mutations were found in all tumor subtypes.

In conclusion, the Class 1 subtype in NMIBC mainly includes tumors with a low grade, a low stage, and a good clinical prognosis. However, Class 3 mainly includes NMIBC in a low proportion and is enriched in stem cell and basal cell markers, similar to the phenotype of CIS (carcinoma *in situ*). Moreover, Class 2 is mainly a highly malignant tumor enriched in uroplakins with a poor clinical prognosis, and the mutated genes are mainly involved in chromatin remodeling.

### High-Risk NMIBC Subtyping

Van Kessel et al. (34) found that the methylation status of *GATA2, TBX2, TBX3*, and *ZIC4* and mutations in *FGFR3, TERT, PIK3CA*, and *RAS* are correlated with the progression of NMIBC (41, 42). Then, the research team detected and analyzed frozen tumor tissues of 1,239 patients with NMIBC from 6 European countries (276 patients were classified as low-risk, 273 as medium-risk and 555 as high-risk according to the EAU guidelines); ultimately, the high-risk NMIBCs were divided into three subtypes.

A total of 57 NMIBCs (4.6%) progressed to MIBCs, with a median follow-up time of 27 months (0–81 months) and a PIR (rate of progression per 100 patients-year) of high-risk NMIBCs = 4.25. Wild-type *FGFR3* and *GATA2* and *TBX3* methylation were significantly correlated with NMIBC progression (HR 0.34, 2.53, and 2.64, respectively). The high-risk NMIBC group was reclassified as a good class (PIR = 0.86, 26.2%), a moderate class (PIR = 4.32, 49.7%), and a poor class (PIR = 7.66, 24.0%) according to *FGFR3* mutations and the *GATA2* methylation status. The classification method was validated and analyzed with another 362 patients who were classified as high-risk NMIBCs according to the EUA guidelines. A total of 30 NMIBCs developed to MIBCs, with a progression rate of 8.3%. All NMIBCs were divided into the good class (95), moderate class (180), or poor class (87). Moreover, 2 of 95 patients in the good class (2.1%), 15 of 180 patients in the moderate class (8.3%), and 13 of 87 patients in the poor class (14.9%) progressed to MIBC. Therefore, this subtyping method combined with the EUA guideline pathological classification system can further classify high-risk NMIBCs into three subtypes with a low, medium and high risk of progression and different clinical prognoses. Therefore, the traditional pathological classification system combined with molecular subtyping greatly improves the prediction ability of the traditional classification system and guides the selection of clinical supervision and treatment schemes.

## Molecular Subtyping Of MIBC

There are an increasing number of molecular subtyping systems on MIBCs, including the UNC (University of North Carolina) two subtyping system, the MDA (MD Anderson) three subtyping system and the TCGA subtyping system. The TCGA database contains a series of genomic, transcriptomic and other data on tumor samples from MIBCs. As the number of patients increased, the subtyping methods developed rapidly, from the original four-subtype system (129 MIBCs) in 2014 to the five-classification system (412 MIBCs) in 2017. Combined with the characteristics of each subtype and its clinical information, the comprehensive analysis provides important information for the diagnosis, treatment and prognosis of bladder cancer.

### UNC Two Subtyping System

Damrauer et al. (31) analyzed 262 high-grade MIBCs by extracting data from four published studies. According to the expression levels related to the differentiation of the urinary epithelium (such as keratin and CD44), MIBCs are divided into two subtypes. As these two molecular subtypes are similar to the expression profiles of the luminal and basal subtypes of breast cancer, they are named luminal-like and basal-like subtypes, respectively, and the basal-like subtype also contains a “claudin-low” subtype.

The disease-specific and overall survival rates of the luminal-like subtype are significantly higher than those of the basal-like subtype (*P*-values are 0.019 and 0.008, respectively). The genome of this subtype is enriched in *FGFR3* (fibroblast growth factor receptor 3) and *TSC1* (tubercular sclerosis 1) mutations, as well as changes in the RB1 pathway, including *RB1* deletion mutations and the amplification of *CCND1* (cyclin D1), *E2F3* (E2F transcription factor 3), and *CCNE1* (cyclin E1). The basal-like and luminal-like subtypes have similar characteristics to breast cancer, and subtypes of breast cancer with claudin-low characteristics can also be found in MIBCs. Moreover, the claudin-low subtype belongs to the basal-like subtype, but there is no significant difference in the disease-specific survival rate or overall survival rate between the two. The claudin-low subtype expresses low levels of claudin-binding protein and high levels of EMT and stem-like marker genes.

### MDA Three Subtyping System

In 2014, a research team from the MD Anderson Cancer Center analyzed the mRNA of 73 MIBC patients' tumors. Their molecular profiles are similar to those of breast cancer and were subtyped as the luminal, p53-like and basal subtypes (35). The luminal subtype is enriched with epithelial markers, activated *PPAR-*γ, estrogen receptor, *FGFR3* mutations, and high *FGFR3* expression, suggesting possible sensitivity to FGFR blockers. The mRNA profiles of the p53-like and luminal subtypes are similar, but the wild-type *p53* gene is significantly activated in the p53-like subtype; therefore, these MIBCs are classified into the p53-like subtype (the TP53 mutation frequency is similar in all subtypes). The p53-like subtype is resistant to neoadjuvant chemotherapy drugs, and all drug-resistant MIBCs become the p53-like subtype after chemotherapy, suggesting that the *p53* gene may play an important role in chemotherapy. Basal MIBCs highly express squamous cell differentiation markers and *p63* and are more invasive and associated with a poor prognosis compared with other subtypes.

Furthermore, the team aimed to determine the relationship among these molecular subtyping systems and others. The Lund 2012 “urobasal A” subtype can be divided into the MDA “luminal” subtype. The Lund 2012 “infiltrated” and MDA “p53-like” subtypes are enriched in extracellular matrix markers. The basal/SCC-like subtype in Lund 2012 and the MDA basal subtype are both enriched in squamous cell differentiation markers. The luminal, p53-like and basal subtypes exist not only in MIBC but also in NMIBC. The luminal subtype is enriched in epithelial markers, showing activated *PPAR-*γ and *FGFR3* mutations, and has a good clinical prognosis. The p53-like subtype belongs to the luminal subtype, but its wild-type *p53* gene is significantly activated and tolerant to chemotherapy. The basal subtype is enriched in squamous cell differentiation markers and activated *p63* and is more invasive and has a poor clinical prognosis.

### TCGA Subtyping System

The Cancer Genome Atlas (TCGA) project was sponsored by The National Cancer Institute and The National Human Genome Institute (USA) to study information related to the human cancer genome. With the increasing number of patients and the development of various detection technologies, biological databases have become increasingly abundant. Therefore, various molecular subtyping systems are being constantly updated and improved. In 2014, the genetic data of 129 MIBCs were analyzed, and it was found that the signature of the molecular subtypes is similar to that of breast cancer, but the name is conservative, and these subtypes were named Cluster I, Cluster II, Cluster III, and Cluster IV (36). Subsequently, TCGA was updated to include 208 MIBCs, which were subtyped into the luminal, immune undifferentiated, luminal immune, and basal subtypes based on their genetic expression signature, which included uroplakins and immune infiltration (43). When the number of MIBCs reaches 412, they can be classified into five subtypes—luminal, luminal-infiltrated, basal-squamous, neural, and luminal-papillary—based on their genetic signature (37).

The TCGA 2014 (36) subtyped MIBCs into Cluster I, II, III, and IV subtypes. Cluster I MIBCs mainly include papilloma pathological phenotypes enriched in *FGFR3* gene mutations; Clusters I and II highly express *GATA3, FOXA1, UPK3A* and luminal and uroplakin genes and are enriched in *RBB2* mutations and estrogen receptor beta (ESR2). Cluster III highly expresses epithelial and precursor or progenitor cells and keratin, and some include squamous cell pathological phenotypes. The most frequent mutations in the MIBC genome are *TP53* (49%), *FGFR3* (12%), *ERCC2* (12%), *RB1* (13%), *CDKN1A* (14%), *EP300* (15%), and *PIK3CA* (20%). The most frequent deletions or amplifications in the genome are *CDKN2A* (47%), *NCOR1* (25%), *YWHAZ* (22%), and *E2F3/SOX4* (20%). Changes in genes or mRNAs involved in the PI3K/mTOR and FGFR3/RTK/RAS signaling pathways may represent potential targets for targeted therapy.

TCGA 2017 (37) then classified 412 MIBCs into five different subtypes: luminal, luminal-infiltrated, basal-squamous, neural, and luminal-papillary. The luminal, luminal-infiltrated, and luminal-papillary subtypes highly express luminal marker genes (*KTT20, GATA3, UPK1A, UPK2, FGFR3*, etc.), and the luminal-infiltrated subtype highly expresses ECM (extracellular matrix) and smooth muscle genes. The luminal-papillary subtype is mainly a papillary pathological phenotype. Basal marker genes (*KRT5, KRT6A, CD44*, etc.), squamous epithelium-like genes (*GSDMC, TGM1*, etc.), and immune marker genes (*CXCL11, L1CAM*, etc.) are highly expressed in basal squamous cells. The neural subtype highly expresses neural differentiation genes.

## BC Molecular Subtyping System

### BOLD 2018 Six Subtyping System

Robertson et al. (38) analyzed the published data of 2411 BCs from TCGA, UROMOL, and IMvigor 210 publication studies, and BCs were reclassified into six subtypes: neural-like (neural 22.7%), luminal-like (LUM 22.7%), papillary-like (PAP 22.7%), HER2-like (HER2L 22.7%), squamous cell carcinoma-like (SCC) (22.7%) and mesenchymal-like (MES 22.7%). Survival analysis was performed on 541 patients with BC. The median overall survival time of the neural, luminal, SCC-like, and mesenchymal subtypes was 87, 91, 107, 20, and 86 months, respectively (the data of the papillary subtype were not available) and that of the six subtypes of 280 MIBCs was 43, 16, 23, 102, 16, and 79 months, respectively. The genes involved in these subtypes are different from those involved in basal, ECM, luminal/uroplakins, immune/infiltration, proliferation, neural differentiation, squamous cell differentiation and other biological processes.

The correlation between BOLD 2018 and the traditional pathological classification showed that the LUM and PAP subtypes are dominant in NMIBCs, while the neural, MES and SCC subtypes are dominant in MIBCs. The proportion of the HER2L subtype in NMIBCs and MIBCs is relatively even. Ta is mainly composed of PAP, while T1 BCs are mainly composed of the LUM and HER2L subtypes, and only a few NMIBCs (20%) are of the neural, MES and SCC subtypes. The HER2L, MES and SCC subtypes are enriched in advanced and high-grade tumors, which are prone to lymph node metastasis and have a poor clinical prognosis.

Moreover, the LUM and HER2L subtypes of NMIBCs have progressive characteristics, while the neural, SCC and MES subtypes of MIBC have progressive characteristics. PAP NMIBCs have a better prognosis and therefore require less supervision. However, the HER2L, LUM, MES and SCC subtypes have a higher risk of progression and therefore require frequent monitoring and aggressive treatment. The tolerance of the MES subtype to chemotherapy is similar to that of the claudin-low subtype. High PD1 and CTLA4 expression signals in the neural, MES and SCC subtypes may be a potential therapeutic target for immune checkpoints.

### Intratumoral Heterogeneity in BC

Molecular subtyping plays an important role in predicting the prognosis and treatment response of bladder cancer patients. However, current subtyping studies are mainly based on detecting the DNA or RNA of tumor tissues or cells. However, traditional sequencing technology is based on whole tumor tissue rather than a single cell; therefore, intratumoral heterogeneity may be an important factor affecting the accuracy of molecular subtyping. Warrick et al. (44) conducted a pathological examination on 309 bladder cancer markers and found that 83 of them exhibited intratumoral variation in BC tumor tissues. Then, these 83 specimens were subtyped with the Lund subtyping system, and 39% exhibited molecular heterogeneity. Eventually, these 83 samples were divided into urothelial-like, genomically unstable, basal-squamous, mesenchymal-like, and neuroendocrine-like subtypes. The basal-squamous subtype shows the greatest variability; approximately 78% of these tumors simultaneously exhibit the genomically unstable or urothelial-like subtype. Heterogeneity in bladder cancer is common, which leads to variation in molecular subtypes and affects the prognosis and treatment response of patients and therefore should be a focus of attention.

## Molecular Subtyping Based On Adjuvant Chemotherapy

### Molecular Subtyping Used to Predict the Response and Prognosis to Neoadjuvant Chemotherapy (NAC)

Seiler et al. (39) used molecular subtyping to predict the response and prognosis to NAC. A total of 343 MIBCs were analyzed by transcriptome sequencing before NAC, and the GSC [single-sample genomic subtyping classifier (GSC)] was developed by comprehensively analyzing the outcomes of NAC BCs in previously published molecular subtyping studies (UNC, MDA, TCGA, Lund, etc.) Then, the MIBCs were divided into four subtypes: claudin-low, basal, luminal, and luminal. Basal tumors were divided into basal and claudin-low subtypes based on the expression of EMT and immune infiltration genes. A comparative analysis of 223 published non-NAC MIBCs' overall survival (OS) found that the predicted accuracy of GSC was 83%. In another 82 validated MIBCs, the accuracy of GSC was 73%, which was significantly higher than that of other major published molecular subtyping systems (37%).

The 3 years overall survival (OS) rate of the GSC basal subtype in the non-NAC group was 49.2% (95% CI 39.5–61.2%; *P* < 0.001) vs. 77.8% (95% CI 67.2–90.0%) in the NAC group; *P* < 0.001). In the non-NAC group, patients with the GSC basal subtype had a risk ratio of 2.22 relative to those with the GSC luminal subtype. The basal and luminal subtypes of GSC in the NAC group were not significantly different. These results indicate that EMT and immune infiltration play an important role in the response to adjuvant chemotherapy and patient prognosis and that patients with the GSC basal subtype benefit from neoadjuvant chemotherapy.

### Adjuvant Chemotherapy Affects Molecular Subtyping

NAC affects the biological signature of a tumor, and the traditional molecular subtyping system is inconsistent before and after NAC treatment. Seiler's research team (40) examined the genes in 133 pre- and post-NAC MIBCs, 116 of which were pre-NAC and post-NAC paired samples. By analyzing the genetic expression signature, cisplatin-resistant bladder cancer patients were divided into four major subtypes: CC1-basal, CC2-luminal, CC3-immune, and CC4-scar-like. NAC does not affect the phenotype of the CC1-basal and CC2-luminal subtypes, which are manifested as a basal and a luminal phenotype pre-NAC, respectively. Basal and luminal markers are lost in the CC3-immune subtype, which is characterized by the highest immune activity after NAC. After NAC, the CC4-scar-like subtype highly expresses wound healing/Scar-related genes, and its prognosis is the best.

Liu et al. (45) conducted full exon sequencing analysis of MIBCs pre- and post-NAC and found that chemotherapy did not increase the overall mutation load of the tumors but increased the mutations of some subclonal tumors. After chemotherapy, these subclonal MIBCs are associated with poor OS, and the genes involved in the cell cycle and immune checkpoint regulation are significantly altered. Cisplatin-based chemotherapy affects the genetic signature of bladder cancer; therefore, studying the altered genes before and after chemotherapy and the effects of these changes may provide important information for the study of chemotherapy resistance mechanisms.

## Discussion

The traditional classification system is based on a comprehensive analysis of the pathological characteristics, recurrence, progression, and prognosis of NMIBCs and then predicts the risk of recurrence and progression of NMIBCs (46, 47). The molecular subtyping system mainly uses cluster analysis to examine the genome and the expression levels of genes and their involved biological processes (25). Compared with the traditional classification system, molecular subtyping reflects the intrinsic characteristics of the tumors, and it not only predicts the prognosis and treatment response of NMIBCs but also MIBCs (48).

Obtaining high-quality samples is the first key step to establish molecular subtyping, but unfortunately, many factors can easily affect the quality of samples, resulting in distortion of the results. NIMBC, especially in the early stage, has a very small tumor size, and it is difficult to obtain enough sample for pathological, genomic, and transcriptometric diagnoses to be performed at the same time. In contrast, the volume of the MIBC tumors is often larger and has enough samples for all of the above testing. This may be one of the reasons why NIMBC molecular typing has developed slowly in recent years, while MIBC molecular typing has developed rapidly. Surgery also affects the quality of tumor samples. Transurethral resection of bladder tumor (TURBT) resects tumor tissue by electrocision, and the length of burning time and the depth of cutting directly affect the quality of tumor tissue. In addition, the longer the operation time is, the more serious the mRNA degradation.

mRNA in resected tumor tissues is easily degraded; a longer cut-off time leads to more serious degradation. However, the surgical time for TURBT or radical cystectomy often varies greatly depending on the operator's experience, so both the surgical method and surgical time are factors that cannot be ignored, as both affect the quality of the specimens. mRNA in frozen samples is relatively stable, while that in formalin-fixed, paraffin-embedded specimens is often severely degraded (49). Because the TME contains a large number of other components, such as stromal cells and immune cells, the inappropriate purification of tissues can also affect the results of molecular subtyping (23). Of course, bladder cancer is a highly heterogeneous tumor, and even if the samples are collected from different sites of the same tumor tissue, its molecular subtyping results may be different (44). Therefore, the establishment of a more reasonable specimen collection and management procedure is the first step to ensure the accuracy of molecular typing results.

### NMIBC Molecular Subtyping

The early molecular classification system subdivided BC into luminal and non-luminal subtypes, mainly according to whether there was high expression of urothelial differentiation biomarkers. Although there are an increasing number of molecular subtyping systems, they are basically built from these two basic subtypes. It should be noted that the basal subtype is just one subtype of the non-luminal type. Due to the high proportion of non-luminal subtype tumors, studies often mistake non-luminal as basal subtype (23). In addition, luminal and basal subtypes are not the exclusive subtypes of MIBC, and these two subtypes also exist in NMIBC. However, it cannot be considered that luminal and basal subtypes in MIBC transform from luminal and basal subtypes in NMIBC, respectively. According to UROMOL 2016, 460 NMIBCs were divided into Class 1 (luminal-like), Class 3 (basal-like) and Class 2 (high-risk luminal-like) subtypes. Class 1 highly expresses luminal-like biomarkers, which is highly similar to urobasal A in the Lund 2012 classification (Figure 3). Class 1 subtypes mainly consist of low-risk NMIBC, while high-risk NMIBC tends to be concentrated in the Class 3 and Class 2 subtypes. Although Class 3 has the characteristics of MIBC basal-like, Jakob Hedegaard thought that Class 3 subtypes could not be considered precursors of MIBC basal-like subtypes. Compared with Class 2 subtypes, the proportion of Class 3 in MIBC was very small, so the team speculated that Class 3 gradually shifted into the Class 2 subtype in progression (33). Second, in addition to the luminal differentiation feature, luminal subtypes highly expressed cell adhesion, early cell cycle, metabolism, and FGFR3 genes. Late cell cycle, CSC markers and EMT-related genes tend to be highly expressed in Class 2 (high-risk luminal-like tumors). In addition, extracellular matrix organization, immune response and other changes were also observed. However, late cell cycle, CSC markers and EMT-related genes tend to be highly expressed in partial non-luminal subtypes; extracellular matrix organization, immune response and other changes were also observed in non-luminal subtypes (32, 33). Third, further research is needed on which genes drive NMIBC to MIBC. Gottfrid Sjödahl's team (50) followed up 357 urothelial bladder cancer patients (UBCs) and analyzed the genes of 73 patients who transformed from NMIBCs to MIBCs. They found that although *FGFR3, PIK3CA*, or *TERT* were the most common mutations of NMIBC, they were not associated with progression, while *TP53* was a common mutation in advanced UBC and highly associated with highly invasive subtypes, suggesting that *TP53* may be a key factor driving the progression of NMIBC (41). The methylation state of *GATA2* may be related to the progression of NMIBC. Van Kessel analyzed the *GATA2* methylation status and *FGFR3* mutation status of high-risk NMIBC and found that NMIBC with *GATA2* methylation and *FGFR3* wild type was more likely to progress into MIBC (34). Although it is not clear which genes drive the transformation of NMIBC into MIBC, molecular typing provides important clues. Molecular subtyping is also helpful to monitor and manage the recurrence and progression of NMIBC, especially high-risk NMIBC. High-risk NMIBC was further classified into good, moderate and poor subtypes, which increased the accuracy of its prediction of the risk of NMIBC progression (34). Therefore, novel molecular subtyping has the potential for implementation in the traditional NMIBC guidelines. As mentioned above, compared with the molecular subtyping of MIBC, the molecular subtyping of NMIBC is developing slowly at present, with relatively few reports in the literature; further study is needed.

### MIBC Molecular Subtyping

During progression, not only the intrinsic characteristics of bladder cancer cells but also the tumor microenvironment (TME) change. Early MIBC molecular classification systems tended to focus on the molecular classification of tumor cells themselves, such as luminal, basal and other subtypes (31, 35). With a deeper understanding of BC cells and their TME, molecular subtyping efforts began to focus more on heterogeneity, the extracellular matrix (ECM) and immune infiltration, promoting further refinement of BC typing. More systematic and comprehensive molecular subtyping has deepened the comprehensive understanding of the characteristics of BC, thus promoting the development of new targeted therapies and immunotherapies in the treatment of bladder cancer. The more systematic and comprehensive molecular subtyping not only deepens the understanding of the characteristics but also promotes the development of novel targeted therapies and immunotherapies in the treatment of bladder cancer. TCGA 2017 subdivided luminal into luminal, luminal-papillary and luminal subtypes and subdivided non-luminal into basal squamous and neural subtypes according to their pathological and genetic characteristics (37). BOLD 2018 also include neural-like, luminal-like, papillary-like, squamous-cell carcinoma-like subtypes and added HER2-like and mesenchymal-like subtypes (38). Recently, Kamoun established and verified a comprehensive network based on six classification systems and divided MIBC into luminal-papillary, luminal non-specified, luminal instability, stroma-rich, basal/squamous, and neurostable-like (51). This classification system considers not only the heterogeneity of BC tumor cells themselves but also the influence of infiltrated immune cells and stromal cells on the clinical characteristics and prognosis of tumors. Therefore, the new subtyping systems partially retain the classical subtypes of the previous subtyping system as well as propose some new subtypes.

First, the prognosis of each subtype is different, and the response to neoadjuvant chemotherapy is also different. Luminal subtypes with relatively mature differentiation tend to have a better prognosis, while basal and neural-like subtypes with poor differentiation have a worse prognosis (23, 33, 38, 51). Although the prognosis of the luminal-papillary subtype is good, it is not sensitive to neoadjuvant chemotherapy (NAC) (37). Although basal/squamous LumNS (luminal non-specified) has a poor prognosis, they may benefit from NAC. In conclusion, not all people can benefit from NAC, and molecular subtyping is beneficial to select patients with specific subtypes that are sensitive to NAC, which could help avoid the toxicity of unnecessary chemotherapy and optimize the therapeutic strategy, a task that is difficult for traditional typing.

Second, based on the comprehensive analysis of the genome, transcriptome, and non-coding RNA of BC, molecular subtyping provides a new direction for personal and precise treatment. Genetic alterations in BC include point mutations, gene fusion, gene amplification, and deletion. The most common genetic changes in NMIBC include *FGFR3, KDM6A, PIK3CA*, and *KMT2D*, while the most common mutations in MIBC include *TP53, KMT2D, KDM6A*, and *RB1*. In addition, APOBEC-mediated mutagenesis may be an important factor in driving genetic mutations in NMIBC and MIBC (1). These altered genes are involved in a number of important biological processes, including TP53/cell cycle, RTK/RAS/PI3K, FGFR3, EGFR, chromatin modifiers, DNA damage, etc. It is the in-depth study of these processes that has promoted the development of BC targeted therapy and accurate diagnostic and treatment approaches. *FGFR3* is one of the most common amplification mutation genes in luminal subtypes, and targeted FGFR has become a promising treatment. FGFR1-4 alterations UC patients respond well to the FGFR inhibitors erdafitinib and rogaratinib (52, 53), and these drugs are undergoing UC II/III clinical trials. Similarly, *PIK3CA* is also a common activated mutant gene, and targeting the PI3K pathway has achieved a good therapeutic effect *in vivo* and *in vitro* preclinical BC treatment (54). In addition to the above targeted therapies, other targeted signaling pathways, such as ERBB2 and TSC1, are undergoing preclinical testing.

Third, molecular subtyping provides guidance for BC immunotherapy. Anti-programmed death 1 (PD-1), programmed death-ligand 1 (PD-L1), and cytotoxic t-lymphocyte antigen 4 (CTLA4) are important second-line therapies for MIBCs with NAC failure. Unfortunately, not all patients can benefit from immunotherapy. The luminal-infiltrated subtype in the TCGA 2017 subtyping system moderately expressed PD-L1 and CTLA-4. In particular, although this subtype is not sensitive to NAC, it has a good response to anti-PD-L1, PD-1 and CTLA-4 treatment. In addition, the basal/squamous subtype is sensitive not only to NAC but also to anti-PD-L1, PD-1, and CTLA-4 (37).

Compared to the traditional BC classification, molecular subtyping provides more information for precise treatment. For example, in the classification system established by Kamoun et al. (51), the luminal papillon (LumP) subtype highly expressed the *FGFR3* gene, and targeted FGFR3 therapy was effective. LumNS (luminal non-specified) and stroma-rich were rich in infiltrated stromal and immune cells. Both of these subtypes highly expressed panfibroblast TGF-β and showed resistance to PD-1 or PD-L1 treatment. Similar to TCGA 2017, the neuroauction (NE) subtype highly expressed cell cycle- and DNA replication-related genes and was sensitive to NAC therapy. The basal/squamous subtype showed good responsiveness to immunotherapy but was not sensitive to radiotherapy.

**Figure 4:**
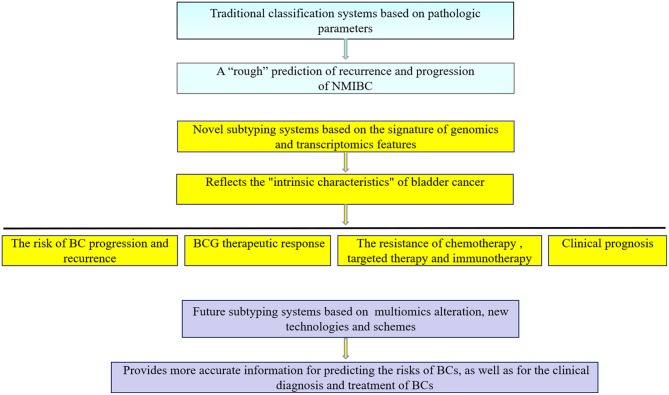
Traditional classification and novel subtyping systems for bladder cancer. NMIBC, Non-muscular invasive bladder cancer; BCG, Bacillus Calmette—Guerin; BCs, bladder cancer patients.

There are an increasing number of molecular subtyping systems that are being constantly updated and provide important information for clinical diagnosis and treatment. However, BC molecular subtyping still has some limitations: (I). In the traditional pathological classification system, it is relatively easy to obtain pathological parameters, it is convenient to operate, and the prediction results are approximate, which is suitable for clinical applications. (II) Molecular subtyping is mainly based on “static” research, especially in NMIBC, and enables a one-time detection and analysis of tumor specimens rather than “dynamic” tracking. For example, during the monitoring and follow-up of NMIBCs, primary, recurrent and progressive tumors should be collected and analyzed to identify a better risk prediction system by integrating the information and data in the whole diagnosis and treatment process. (III) Currently, molecular subtyping is mainly focused on genome and transcriptome research, whereas the proteomics and immune status of tumors are also closely related to their development. Therefore, multiomics should be included in the study of molecular subtyping. (IV) Bladder cancer often exhibits intratumor heterogeneity. There may be multiple molecular subtypes in the same tumor, which may be an important factor affecting the accuracy of the molecular subtyping prediction system. With the rapid development of single-cell high-throughput sequencing (46, 55, 56), mass spectrometric detection (57), immune analysis (58, 59), and other technologies, the accuracy of the molecular subtyping prediction system will be further improved (Figure 4). Compared with traditional pathological classification, molecular subtyping offers a more comprehensive analysis of the genome, transcriptome and non-coding RNA, providing important guidance for BC adjuvant chemotherapy, targeted therapy and immunotherapy. In the future, these classifications will become an important complementary approach to traditional pathological classification.

## Author Contributions

SZ and WY drafted the manuscript. FC, XY, and CW revised the content and approved the manuscript for publication.

### Conflict of Interest

The authors declare that the research was conducted in the absence of any commercial or financial relationships that could be construed as a potential conflict of interest.
